# 2D/1D V_2_O_5_ Nanoplates Anchored Carbon Nanofibers as Efficient Separator Interlayer for Highly Stable Lithium–Sulfur Battery

**DOI:** 10.3390/nano10040705

**Published:** 2020-04-08

**Authors:** Zongtao Zhang, Guodong Wu, Haipeng Ji, Deliang Chen, Dengchao Xia, Keke Gao, Jianfei Xu, Bin Mao, Shasha Yi, Liying Zhang, Yu Wang, Ying Zhou, Litao Kang, Yanfeng Gao

**Affiliations:** 1 School of Materials Science and Engineering, Zhengzhou University, Kexue Ave 100, Zhengzhou 450001, China; wuguodong000@163.com (G.W.); jihp@zzu.edu.cn (H.J.); dlchen@zzu.edu.cn (D.C.); xdc19941001@163.com (D.X.); gkk15837400326@163.com (K.G.); xu18238727241@163.com (J.X.); mao290352192@163.com (B.M.); yiss@zzu.edu.cn (S.Y.); zhangliying@zzu.edu.cn (L.Z.); wangyu@zzu.edu.cn (Y.W.); zhouying@zzu.edu.cn (Y.Z.); 2 College of Environment and Materials Engineering, Yantai University, Yantai 264005, China; 3 School of Materials Science and Engineering, Shanghai University, Shangda Rd 99, Shanghai 200444, China

**Keywords:** V_2_O_5_ nanoplates, carbon nanofiber, quasi-confined cushion space, interlayer, lithium–sulfur batteries

## Abstract

Quick capacity loss due to the polysulfide shuttle effects is a critical challenge for high-performance lithium–sulfur (Li–S) batteries. Herein, a novel 2D/1D V_2_O_5_ nanoplates anchored carbon nanofiber (V-CF) interlayer coated on standard polypropylene (PP) separator is constructed, and a stabilization mechanism derived from a quasi-confined cushion space (QCCS) that can flexibly accommodate the polysulfide utilization is demonstrated. The incorporation of the V-CF interlayer ensures stable electron and ion pathway, and significantly enhanced long-term cycling performances are obtained. A Li–S battery assembled with the V-CF membrane exhibited a high initial capacity of 1140.8 mAh·g^−1^ and a reversed capacitance of 1110.2 mAh·g^−1^ after 100 cycles at 0.2 C. A high reversible capacity of 887.2 mAh·g^−1^ is also maintained after 500 cycles at 1 C, reaching an ultra-low decay rate of 0.0093% per cycle. The excellent electrochemical properties, especially the long-term cycling stability, can offer a promising designer protocol for developing highly stable Li–S batteries by introducing well-designed fine architectures to the separator.

## 1. Introduction

Fast development of portable electronic devices and electric vehicles calls for battery with high energy density and long-lasting cycle performance [1,2]. With the state-of-the-art Li-ion batteries approaching their theoretical limits, lithium–sulfur (Li–S) batteries have been attracting increasing attentions as one of the most promising high-energy-density rechargeable battery systems [3]. Li–S battery can offer a high theoretical capacity of 2567 Wh·kg^−1^ (the Gibbs energy of the Li/S reaction) [4,5,6], and sulfur is relatively low cost and abundant in nature, which are crucial for the future large-scale applications [7,8,9].

However, practical applications of Li–S batteries remain challenge hindered by several undermining issues such as the highly insulating nature of elemental sulfur (S_8_) (5 × 10^−30^ S·cm^−1^ at 25 °C) [10], the large volumetric changes of sulfur during charge and discharge (≈79%) [11,12], as well as the dissolution and shuttling of the lithium polysulfide intermediates (LiPSs, Li_2_S*_x_*, 4 ≤ *x* ≤ 8), which result in low utilization and loss of cathode active materials [13]. Massive efforts, including modification of the sulfur cathode, compositional design for the electrolyte, functionalization of the separator, and protection of the active Li anodes, have been devoted to manipulate these deficiencies [14,15,16,17,18,19], e.g., various host materials, including doped carbon and its derivatives [20], metal oxides [21], sulfides [22], and polymers [23], are applied to enhance the chemical or physical affinity with the polysulfides to entrap them within the cathode. Other compounds, like CoS_2_ [24], MoO_3_ [25], and CoP [26], can both catalyze the electrochemical conversion of the polysulfides and adsorb them, which are attractive for polysulfide immobilizing in both cathode and separator parts. Quite recently, insertion of membranes (or the so-called interlayers) between the separator and cathode has been demonstrated as a simple and promising approach to alleviate these issues [27,28]. First, the functional layer can serve as a physical barrier to intercept the migration of the soluble LiPSs to the anode side. Additionally, during the discharge–charge process, the interlayer can help reuse the soluble LiPSs, which thus works as a secondary current collector and enhances the utilization of the active materials [29]. Carbon nanotube (CNT)-based conductive porous materials are the most frequently reported interlayers that were applied in Li–S batteries [30]. However, due to the weak interactions between the nonpolar carbon matrix and the polysulfides, the enhancements from these interlayers are still deficient for practical applications [31,32]. Progressively, metal oxides, like TiO_2_ [33], MnO_2_ [34], and Fe_3_O_4_ [35], etc., have demonstrated the strong adsorption attractions with the LiPSs, by virtue of the intense electrostatic attractions between the metal−oxygen bond and the LiPSs. However, there are still gradual declines of the specific capacity after long-term cycling, especially due to the formation of inactive sulfur species inside the interlayer, which impede the ion pathway and/or increase the interfacial impedance between the electrode and the electrolyte. This influence can become worse when cells are assembled with high S loading content or cycling at a high current density. Further steps on separator interlayers are critically needed to establish stable electron and ion pathway while retaining efficient LiPSs trapping for high-performance Li–S battery.

V_2_O_5_ is a well-known layer-structured transition metal oxide that is widely applied in energy storage fields [36,37,38]. Various V_2_O_5_ architectures such as nanorod, nanofiber, nanosphere, nanoflower, nanotube, etc., have been developed for applications in Li-ion batteries [39,40,41,42]. Besides, in these years, there were several contributions of V_2_O_5_ as the sulfur cathode modification materials [43], where catalytic effect and strong adsorption with the LiPSs were observed [44,45]. Recently, investigations from Guan et al. even demonstrated that V_2_O_5_ possess a higher LiPSs adsorption content than the conventionally reported MoS_2_, FeS, CoS, and Ti_4_O_7_ [46]. However, as encountered by other cathode modification materials, specific energy loss (as high as 20–50%) are also accompanied by the incorporation of these inactive host materials. From another perspective, Liu et al. [47] reported that V_2_O_5_-coated carbon membrane can work as an effective interlayer that significantly suppresses the LiPSs shuttling and prompts a superior electrochemical performance. It is quite expected that V_2_O_5_ can be of much more potentials acting as an interlayer material other than the cathode modification materials in Li–S battery. However, as reported by Liu et al. [47], the semiconductive V_2_O_5_ increased the interfacial impedance after direct coating on the carbon surface, resulting in a relatively weak capacitance retention especially when cycling at a high current density. A designer tactic, with considerations of the efficient LiPSs utilization and stable electron and ion pathway building, is required, to fulfill the benefits of V_2_O_5_ as an effective interlayer modification material in Li–S battery.

In these regards, we present here a 2D/1D V_2_O_5_ nanoplates anchored CF networks as an effective separator interlayer for highly stable Li–S batteries. The CF was prepared by direct carbonization of the electrospun polyacrylonitrile (PAN) membrane, and the near-vertically aligned V_2_O_5_ nanoplates on the CF were obtained via a simple hydrothermal process. The specially aligned V_2_O_5_ nanoplates on the CF provides a quasi-confined cushion space (QCCS) that flexibly adsorbs and intercepts the soluble LiPSs. Here, an architecture-derived mechanism for building stable ion and electron transportation pathways was proposed. The corresponding Li–S battery assembled with the V-CF decorated PP interlayer exhibited a high initial capacity of 1140.8 mAh·g^−1^ and a stable reversed capacitance of 1110.2 mAh·g^−1^ after 100 cycles at 0.2 C, and a high capacity retention of 95.4% after 500 cycles (with a superior low decay rate of 0.0093% per cycle) at 1 C was also demonstrated. These achievements, especially the high retention of the capacity for long-term cycling, are quite high among the emerging high-performance Li–S batteries, which may provide a great potential for construction of highly stable batteries.

## 2. Experimental

### 2.1. Raw Chemicals

All chemicals were commercially available and used as received. Polyacrylonitrile (PAN) (*M*_w_ = 150,000) and polyvinyl pyrrolidone (PVP) were purchased from Sigma-Aldrich Co., Ltd., Shanghai, China. N, N-Dimethylformamide (DMF) was purchased from Sinopharm Chemical Reagent Co., Ltd., Shanghai, China. 

### 2.2. Preparation of CFs

The carbon fibers were synthesized by electrospinning. Typically, a precursor solution was prepared by dissolving 0.4 g PAN in 5 mL DMF. Then, the above solution was electrospun onto the target rotating collector (aluminum foil, located 10–20 cm from the syringe needle) under a fixed voltage of 16 kV. After that, the as-obtained PAN fibers were peeled off from the collector and placed in a drying oven at 60 °C to remove the residual DMF. Finally, the PAN nanofibers were peroxidized by annealing in air at 280 °C for 1 h, and carbonized at 900 °C for 2 h under flowing N_2_ atmosphere.

### 2.3. Preparation of V_2_O_5_ Nanoplates Decorated CF

**Figure 1:**
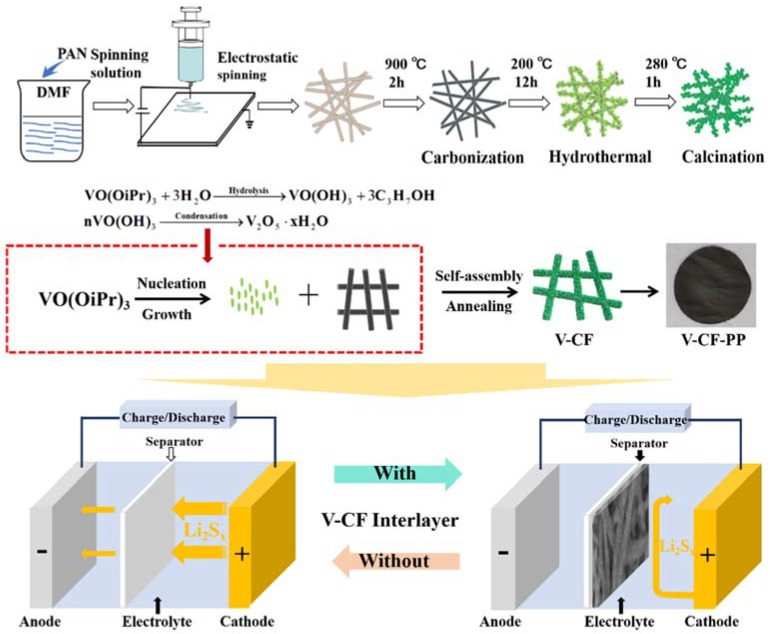
Schematic diagram for the synthesis of 2D/1D V_2_O_5_ nanoplates anchored carbon nanofiber (V-CF) composite interlayer. A photograph of the V-CF interlayer coated on polypropylene (PP) separator is also shown. The bottom two figures show the schematic configurations of the Li−S cells with (right figure) and without (left figure) the V-CF separator interlayer.

The V_2_O_5_ was deposited onto the CF by a hydrothermal method [48,49]. Briefly, 0.2 mL of vanadium (V) oxytriisopropoxide was added into 50 mL isopropanol under stirring. Subsequently, the above-prepared solution (in which *Φ*-19 mm CFs were immersed) was transferred to a Teflon-lined stainless-steel autoclave, sealed, and kept at 200 °C for 12 h. The as-obtained membrane was taken out, rinsed in ethanol and dried under vacuum at 60 °C overnight, which was finally converted into V-CF by annealing at 280 °C for 1 h in air. The schematic diagram for the synthesis of V-CF is shown in Figure 1.

### 2.4. Battery Fabrication and Electrochemical Measurements

The cathodes were prepared by mixing the sulfur powder with the grounded carbon fibers as reported in ref [50], which were annealed at 200 °C for 0.5 h under vacuum in a tube furnace. A viscous slurry was prepared by mixing 80 wt.% active materials, 10 wt.% conductive carbon black, and 10 wt.% poly (vinylidene fluoride) (PVDF) in N-methyl-l-2-pyrrolidone (NMP) dispersant. The slurry was casted on an aluminum foil and dried in vacuum at 60 °C for overnight. The electrolyte consisted of 1 mol·L^−1^ bis(trifluoromethane) sulfonimide lithium salt (LiTFSI) which was dissolved in a mixture of 1,3-dioxolane (DOL) and dimethoxyethane (DME) (1:1 by volume) with 1 wt.% LiNO_3_. The Li–S batteries were assembled into 2032-type coin cells in an argon-filled glove box (O_2_ and H_2_O < 0.1 ppm) with lithium foil as the counter electrode. PP, CF interlayer modified PP (CF-PP), and V-CF interlayer modified PP (V-CF-PP) were applied as the separators for the corresponding cells.

### 2.5. Saturated Adsorption Content Tests

The content of V_2_O_5_ in V-CF was firstly calculated by measuring the weight loss of the oven-dried V-CF samples before and after removing V_2_O_5_ in nitric acid solution. For the saturated adsorption content measurement, about 2 mg V-CF sample was added into the sample bottle and stirred with 10 mL electrolyte; then, diluted Li_2_S_6_ solution (5 mmol·L^−1^ in electrolyte) was added slowly, with a 30 min stirring for each one to two drops. The solution evolved quickly from colorless to light yellow after addition of the deep yellow-colored Li_2_S_6_ solution, and gradually changed back to colorless because of the V_2_O_5_ adsorption. A saturated adsorption state can be obtained when the color of the solution cannot change back after a certain amount of Li_2_S_6_ addition, where the saturated adsorption content of the V-CF can be calculated.

### 2.6. Electrochemical Characterization

The above-fabricated Li–S batteries were cycled between 1.7 and 2.8 V at different current rates on a battery analyzer (Land CT 2001A, Landian, Wuhan, China) at room temperature. The cyclic voltammetry (CV) was tested on an electrochemical workstation (PGSTAT302N, Metrohm, Switzerland) at a scan rate of 0.1 mV·s^−1^ between 1.7 and 2.8 V. The specific charge and discharge capacities were calculated based on the mass of the elemental sulfur. The electrochemical impedance spectra (EIS) of the working electrodes were recorded on the electrochemical workstation in a two-electrode configuration with frequency ranging from 10^−2^ to 10^5^ Hz.

Symmetric batteries were also assembled to investigate the catalytic properties, by placing two of the V-CF (or CF) electrodes into a standard 2032-type coin cell with Celgard 2400 membrane as the separator and 80.0 μL Li_2_S_6_ as the active material. The CV measurements of the symmetric batteries were operated on an electrochemical workstation at a scan rate of 50 mV·s^−1^ between −0.8 and 0.8 V.

### 2.7. Characterization

The morphologies and structure of the CF and V-CF were characterized by scanning electron microscopy (SEM) (Auriga FIB, Zeiss, Germany) and transmission electron microscope (TEM) (Tecnai G220, Hillsboro, OR, USA). The X-ray photoelectron spectroscopy (XPS) spectra was recorded on a PHI Quantera SXM (ULVAC-PHI, Kanagawa, Japan) system with Al/K anode (photon energy = 1486.6 eV) mono X-ray source. Powder X-ray diffraction (XRD) pattern was collected on an X-ray diffractometer (DX-2700BH, Dandong Haoyuan, Liaoning, China) using Cu *K*_α_ as the radiation source. Raman spectroscopy was measured by a Confotec MR520 instrument (Graben, Germany) with an excitation wavelength of 532 nm. Thermogravimetric (TG) analysis was performed under nitrogen flow using a thermal analyzer apparatus (STA449F3, NETZSCH, Germany) with heating rate of 10 °C·min^−1^ from room temperature to 800 °C. Nitrogen adsorption–desorption isotherms were recorded on an ASAP 2460 (Micromeritics Shanghai, China) apparatus at temperature of 77 K. The specific surface area and the pore structure were measured by the nitrogen sorption using a physisorption analyzer (JW-BK112, Beijing, China). The zeta potential was conducted by a Zeta potentiometer (JS94H, Shanghai, China). 

## 3. Results and Discussion

**Figure 2:**
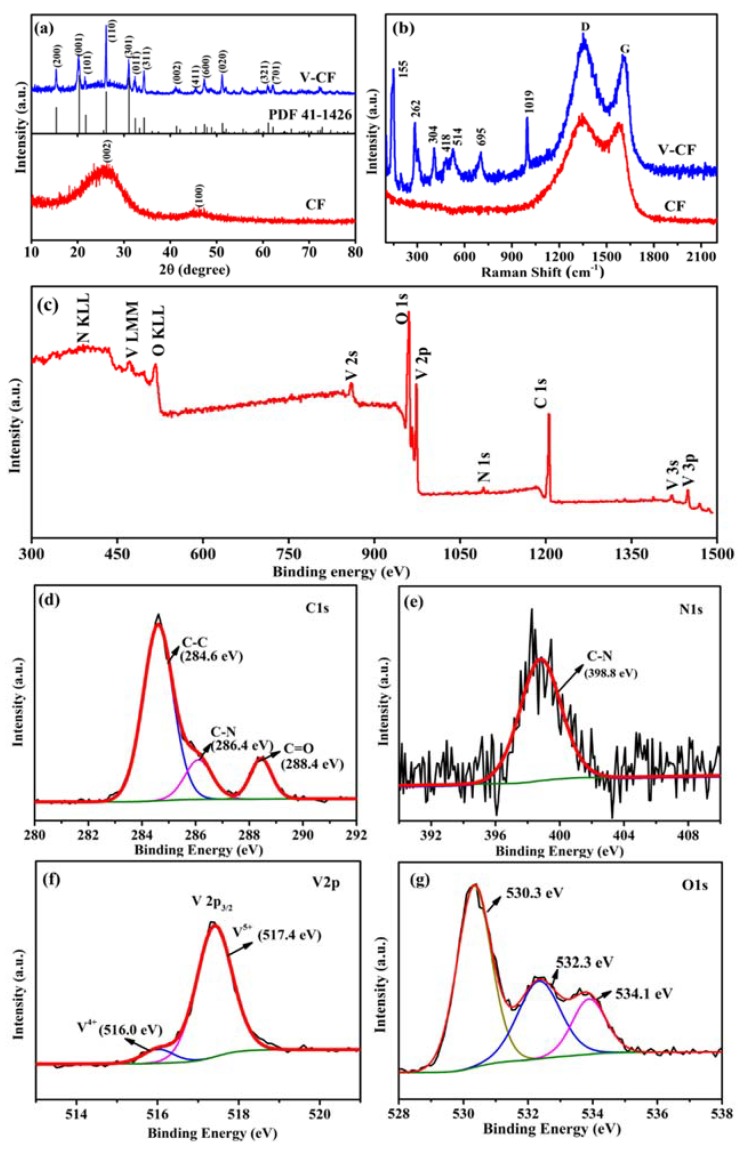
XRD patterns (**a**) and Raman spectra (**b**) of the carbon nanofiber (CF) and the V-CF. XPS wide-range survey spectrum (**c**) and the high-resolution XPS spectra of C 1s (**d**), V 2p (**e**), N 1s (**f**), and O 1s (**g**) for the sample of V-CF.

The XRD patterns and Raman spectra were collected to investigate the structure and composition of the CF and the V-CF samples (Figure 2). The standard diffraction pattern for the orthorhombic V_2_O_5_ (JCPDS Card No.: 41-1426, space group of *Pmmn* (59), a = 11.5 Å, b = 3.6 Å, and c = 4.4 Å) was also given as reference [51]. According to the XRD results, there were two broad peaks centered at around 25.0° and 43.2° for the CF, which can be indexed to the (002) and (100) diffractions of crystalline carbon [52,53]. For the V-CF, all the recorded XRD peaks can be assigned to the orthorhombic V_2_O_5_, without detections of any other vanadium oxides within the accuracy of measurements. Raman spectra further showed the existence of two broad modes centered at 1365 and 1595 cm^−1^ for the CF sample, which can be assigned to the D and G bands for carbon, respectively [54]. For the V-CF, Raman modes centered at 155, 262, 304, 418, 514, 695, and 1019 cm^−1^, were observed, which can be assigned to the orthorhombic phase V_2_O_5_ [55,56]. All these measurements indicated the formation of orthorhombic V_2_O_5_ and carbon in the V-CF. Moreover, X-ray photoelectron spectroscopy (XPS) was performed to investigate the chemical state of the V-CF (Figure 2c–f). Wide-range survey XPS spectrum suggested the existence of carbon, vanadium, oxygen, and nitrogen signals in the V-CF. The fitted pattern for the C1s spectrum indicated the existence of three different profiles of carbon with binding energies of 284.6, 286.4, and 288.4 eV, corresponding to the chemical bonds of C-C [57], C-N [58], and C=O [59], respectively. For the N1s spectrum, a binding energy of 398.8 eV, corresponding to the pyridinic-type N in carbon layer [60], was found, indicating the formation of N-doped carbon structure in the V-CF. Moreover, the fitted curves for the V2p spectrum (Figure 2e) indicated that there were two kinds of vanadium valence states with binding energies of 517.4 eV and 516.0 eV, respectively, which can be assigned to the dominant pentavalent vanadium bonds in V_2_O_5_ and small amount of V^4+^ defect in the sample [61,62,63,64]. For the O 1s spectrum (Figure 2g), three individual peaks, centered at 530.3, 532.3, and 534.1 eV, were found from the fitted XPS curves, which can be attributed to the binding energies of V-O-V in V_2_O_5_ [65], V-OH [66], and C-O [67], respectively. The latter two signals are probably components from the surface contaminating groups for V_2_O_5_ and carbon fibers.

**Figure 3:**
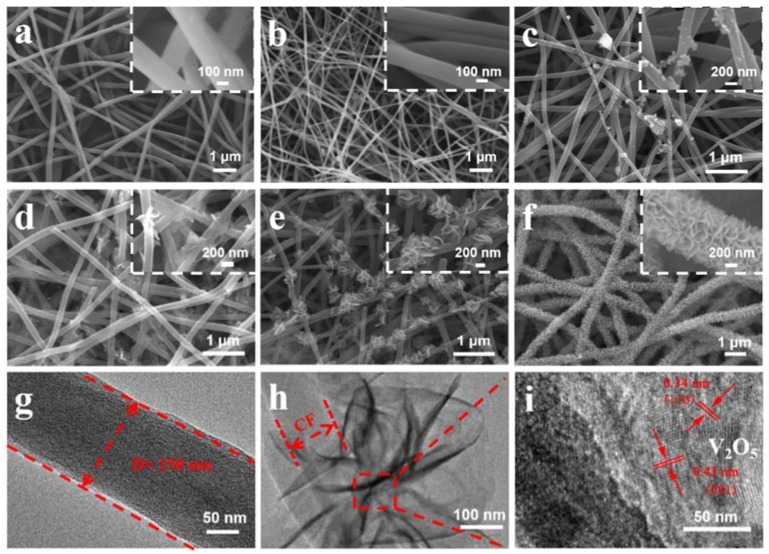
SEM images of the precursor polyacrylonitrile (PAN) fibers before (**a**) and after (**b**) carbonization. Figure (**c**)–(**f**) shows the SEM images for the CF membrane hydrothermally treated at 200 °C for 1, 4, 8, and 12 h, respectively. (The inset of each figure shows the corresponding high magnification images). (**g**) shows the TEM image for CF sample, and (**h**) and (**i**) are the TEM and HRTEM images, respectively, for the V-CF.

The morphologies and structure for the electrospun PAN, the carbonized PAN fibers (sample CF), and the V_2_O_5_ anchored carbon fibers (sample of V-CF) were characterized by SEM and TEM (Figure 3). The pristine electrospun PAN displayed quite porous structures with cross-linked thin fibers (diameter of 150–250 nm) (Figure 3a). After carbonization at 900 °C for 2 h, the main porous structures were retained, although the fiber diameter was shrunk slightly to be around 100–200 nm (Figure 3b). For the V-CF sample, after deposition of V_2_O_5_, a significant morphology change, especially on surfaces of the carbon fiber, was observed. A gradual evolution from small dots, to quasi-isolated nanoplates, and further, to uniformly assembled nanoplate arrays on the fibers, can be clearly recognized, with a prolonged hydrothermal treating time (Figure 3c–f). TEM (Figure 3g,h) and HRTEM (Figure 3i) further showed that these nanoplates can be assigned to orthorhombic V_2_O_5_, which were near-vertically grown on surfaces of the CF. This structure is quite different from the structure as reported in the study of Liu et al. [47], where uniform coating of V_2_O_5_ on the CF was obtained. However, this may derive from a varied hydrothermal condition and/or the different structures of carbon fiber applied. As per literatures, vanadium (V) oxytriisopropoxide can hydrolyze into vanadium oxytrihydroxide, which further nucleate and precipitate to form sheet-like V_2_O_5_·H_2_O (a reaction scheme is shown in Figure 1). The existence of surface functional groups or defects, such as the carbonyl group and the pyridinic-type N in carbon as confirmed in the XPS, can serve as effective nucleation centers for the growth of V_2_O_5_·H_2_O, [51] which can help induce the growth of sheet-like V_2_O_5_ on surfaces of the CF. Similar growth model of near-vertically aligned V_2_O_5_ nanoplates on carbon substrate were also reported from a similar hydrothermal condition, [68] which further supported our results for the growth of the 2D/1D V_2_O_5_ nanoplates on the CF structures for the V-CF. Moreover, it is worth noting that this special 2D/1D structure is expected to afford more promising benefits than the reported uniform thin V_2_O_5_ coating on the carbon fiber, which will be discussed in the following parts.

**Figure 4:**
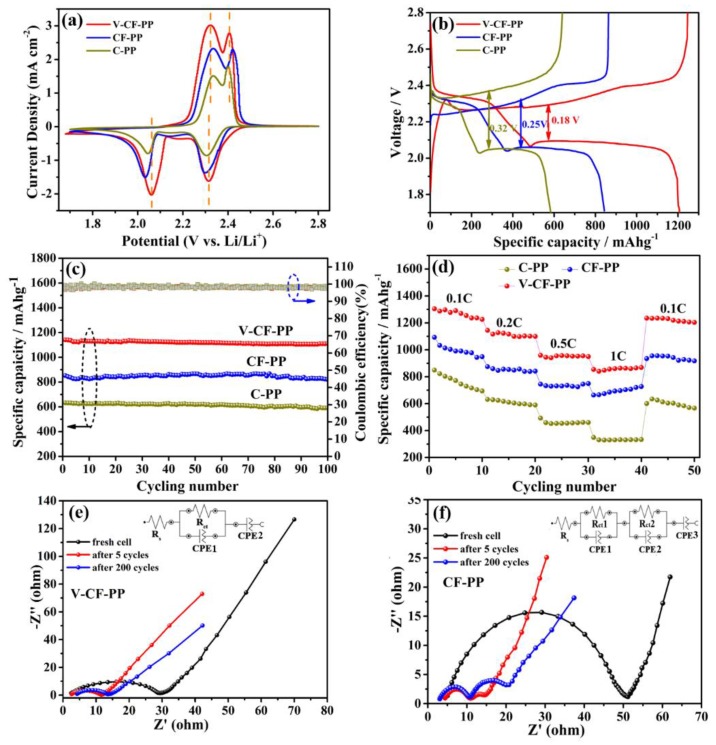
Electrochemical characterizations for the assembled batteries with PP, CF modified PP, and V-CF modified PP as the separators. (**a**) The second cycle cyclic voltammetry (CV) curves measured at a scan rate of 0.1 mV·s^−1^. (**b**) The charge/discharge profiles measured at a current rate of 0.2 C. (**c**) The capacity and coulombic efficiencies versus the cycle numbers measured at a current rate of 0.2 C. (**d**) The rate performance for different cells measured at 0.1, 0.2, 0.5, and 1 C, respectively. (**e**) and (**f**) are the electrochemical impedance spectroscopy (EIS) plots for the CF-PP and the V-CF-PP cells, respectively, measured at different cycles. (The inset of figure shows the equivalent circuit diagram of the CF-PP and V-CF-PP after 200 cycles.).

**Figure 5:**
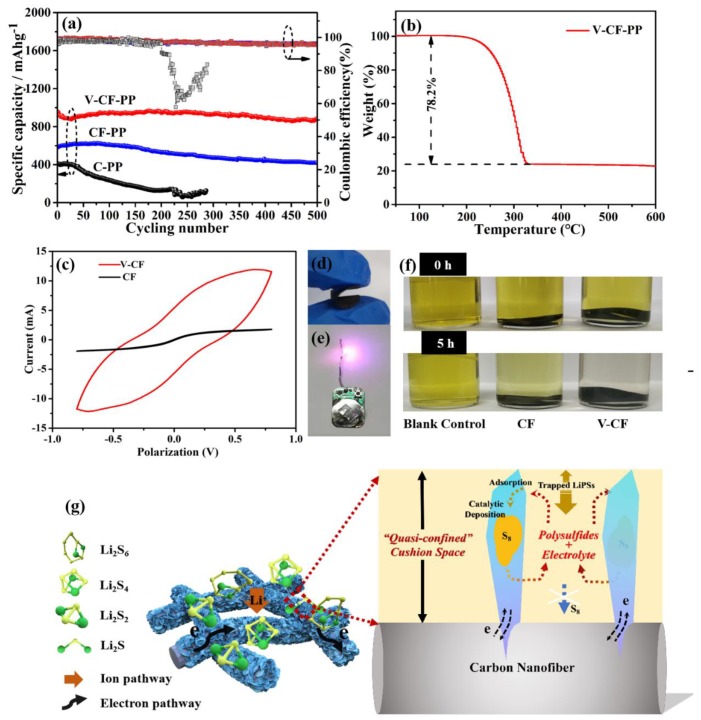
(**a**) Long-term cycling performance at 1 C for different batteries, (**b**) thermogravimetric (TG) analysis of the sulfur loadings in CF/S cathodes, (**c**) CV of symmetric cells using the CF and the V-CF membranes as the corresponding symmetric electrodes, (**d**) the flexibility test of the V-CF interlayer, (**e**) V-CF-PP battery lighting the diode, (**f**) photographs show the adsorption of Li_2_S_6_ in 1 M LiTFSI of 1,3-dioxolane (DOL)/dimethoxyethane (DME) electrolyte solution, and (**g**) the illustration for the cycling stability derived from a quasi-confined cushion space in the V-CF interlayer.

The electrochemical performances for lithium–sulfur batteries assembled with PP, CF modified PP, and V-CF modified PP as the separators, are shown in Figure 4. In the cyclic voltammetry (CV) curves (Figure 4a), two pairs of distinct redox peaks, indicating the reactions among sulfur (S_8_), long-chain lithium polysulfides (Li_2_S*_x_*, 3 ≤ *x* ≤ 8), and the insoluble short-chain Li_2_S_2_/Li_2_S [69,70,71], were observed. Interestingly, at the high sulfur loading content of about 78.2 wt.% (see the TG result, Figure 5b) applied in our investigation, the V-CF-PP cell still showed a quite low polarization, with a voltage gap of only about 0.18 V. The recorded peak current for the V-CF-modified battery was also the highest among the three samples, indicating a significantly increased polysulfide redox kinetics. Figure 4c shows the cycling performance of the three Li–S batteries after charging/discharging for over 100 cycles at 0.2 C. In a detail, the C-PP cell showed an initial capacity of 632.3 mAh·g^−1^, which further decreased to be around 590.5 mAh·g^−1^ after 100 cycles; for batteries assembled with the V-CF-PP and CF-PP separators, high initial capacities of 1140.8 and 852.2 mAh·g^−1^, and excellent residual capacity of 1110.2 and 820.6 mAh·g^−1^ (corresponding to a loss of only 0.31 and 0.32 mAh·g^−1^ for each cycle) after 100 cycles, were delivered. These values, especially for the V_2_O_5_ deposited sample of V-CF-PP, were quite noteworthy among the reports for Li–S batteries. Rate performance, measured from 0.1 to 1 C (Figure 4d), also clearly showed the advantages of the V-CF-PP separator over the CF-PP or C-PP counterparts. In details, the specific discharge capacities of the V-CF-PP cell were 1268.2, 1112.9, 952.3, and 858.2 mAh·g^−1^, respectively, at current densities of 0.1, 0.2, 0.5, and 1 C. The corresponding capacity of the CF-PP and C-PP cells were only 997.6, 851.8, 735.6, and 693.8 mAh·g^−1^ and 762.6, 610.0, 460.2, and 333.2 mAh·g^−1^, respectively. When switched back to 0.1 C again, the V-CF-PP cells recovered about 96.2% of its initial capacity, indicating the stable and highly reversible properties of battery obtained by the V-CF separators. 

Electrochemical impedance spectroscopy (EIS) analysis was also performed to investigate the charge-transferring properties for the CF-PP and the V-CF-PP, taken for the fresh cell, fully charged cell after 5 cycles, and after 200 cycles, respectively. We can see from the EIS plots that the impedance of the V-CF-PP and the CF-PP cells decreased after the first five cycles, due to the utilization of the conductive network after the redistribution of the active materials in interlayers and cathode induced by the shuttle phenomenon [72]. For the CF-PP cell, additional semicircle locating at the high frequency region (around 10–20 Ω) appeared in the Nyquist plot, which can be assigned to the formation of additional resistive phase (e.g., irreversible precipitates of solid Li_2_S and Li_2_S_2_) on the CF networks during cycling [73,74]. After long-term cycling for 200 times, the impedance for this part increased unwillingly, indicating an adverse accumulation of resistive phases that may block interfacial charge transferring. Correspondingly, for the V-CF-PP cell, a single charge-transfer resistance (around 2–10 Ω) was observed after 5 activation cycles, which still retained after 200 cycles of charging/discharging, indicating that the charge transfer across the electrode–electrolyte interfaces for the V-CF interlayer were quite stable. The inset images showed the equivalent circuits of the two samples, which were composed of the solution resistance (Rs), the charge-transfer resistance (Rct), and the double-layer capacitance (CPE). After long-term cycling for CF-PP cell, additional impedances, corresponding to Rct2 and CPE2, were introduced.

Long-term cycling at a higher charging and discharging rate of 1 C was performed to investigate the cycling stability for the three batteries (sulfur area loading amount of about 1.6 mg·cm^−2^) (Figure 5a). We can see from Figure 5a that the V-CF-PP cell delivered an initial capacity of 930.4 mAh·g^−1^ and a reversible capacity of 887.2 mAh·g^−1^ after 500 cycles (capacity retention rate of 95.4%), which are much better than the CF-PP and the C-PP cells. A purple LED powered by the V-CF-PP cell was also used (in Figure 5e), showing the practical potentials as portable power supply for LED lighting. The calculated decay rate was about 0.0093% per cycle for the V-CF-PP cell, which was an ultra-low value compared with literature-reported volumes, especially for different interlayers-modified lithium–sulfur batteries, e.g., 0.09% per cycle for mesoporous TiN (400 cycles at 1 C) [74], 0.083% per cycle for MoS_2_ nanosheets (600 cycles at 0.5 C) [75], and 0.07% per cycle for MTO-CNTs (500 cycles at 0.5 C) [76]. This high-capacity retention ability, especially the long-term stability at high charge–discharge rate, is expected to be originated from the special structures of 2D/1D V_2_O_5_ nanoplates on CF networks, which can serve as an effective quasi-confined cushion space (QCCS) that prompt the construction of stable electron/ion transportation pathway while maintaining excellent LiPSs interceptions. In addition, the V-CF membrane can be folded nearly in half (Figure 5d), demonstrating a good flexibility and mechanical toughness.

The schematic diagram for the V-CF interlayer-modified cell, Figure 5g, shows that there are several benefits for the specially aligned V_2_O_5_ 2D structures on the conductive CF networks. First, the interconnected carbon fiber networks can provide effective electron conductivity that is helpful for high current density and fast redox kinetics. Second, as V_2_O_5_ is one of the high-adsorption-content materials for the LiPSs, [77] the vertically aligned V_2_O_5_ nanoplates on CF can further allow more adsorption sites to endorse LiPSs stabilization. The adsorption experiment in Figure 5f shows that, after 5 h immersion in the Li_2_S_6_ solution, the bottle containing V-CF appears almost transparent, while the others still show certain yellowish color. For the saturated adsorption test as described in the experimental section, a high saturated adsorption content of 35.2 μmol·cm^−2^ was obtained for the V-CF sample, which is much higher than the reported results of 22.3 μmol·cm^−2^ for V_2_O_5_ powders [78]. Third, catalytic-related contributions from the V_2_O_5_ should also be considered to improve the stability. Figure 5c shows the catalytic experiments conducted on symmetrical cells assembled with the CF and the V-CF electrodes, respectively. A significantly enhanced redox current for the V-CF symmetric cell than the bare CF control cell can be observed under a polarization from −0.8 to 0.8 V, which implied a higher catalytic property of V_2_O_5_ toward the LiPSs redox. In this case, a direct deposition of the solid-state sulfur species (e.g., S_8_ and Li_2_S) on V_2_O_5_ nanoplates, instead of forming them on the conductive carbon fibers, is expected, which can suppress the increasement of impedance during cycling.

**Figure 6:**
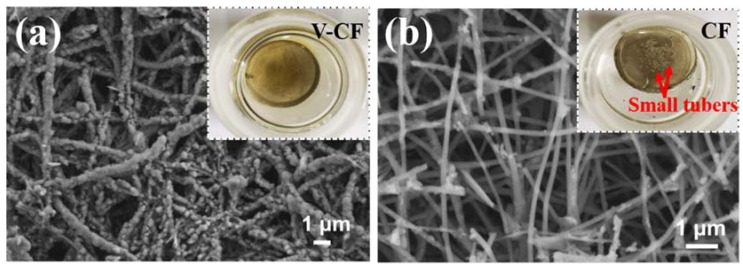
SEM of the V-CF (**a**) and the CF (**b**) interlayers after 100 cycles. Insert in each figure shows the photographs of the corresponding lithium electrode after 100 cycles at 1 C. The metallic lithium electrodes for both samples are immediately immersed into a mixture of DOL and DME for photograph observation.

Moreover, after the formation of the soluble long-chained LiPSs from the first discharging cycle and their shuttling between the anode and the cathode in the electrolyte, more and more LiPSs will be adsorbed and stored into the QCCS on the surfaces of carbon fiber (Figure 5g). Because of the catalytic redox-related deposition and the large saturated adsorption content of the vertically aligned V_2_O_5_ nanoplates, most of the LiPSs compounds will be trapped inside the QCCS, which is especially favorable for building stable ion transportation pathways in Li–S batteries. SEM images for the detached interlayers from the V-CF and the CF cells after 100 cycles discharge and charge at 1 C were further employed to investigate their morphology evolution after long-term cycling. These results are shown in Figure 6. Figure 6 shows large amounts of sulfur species (most possibly in the form of S_8_ at the fully charge state) around the fibers in the V-CF interlayer, demonstrating the successful adsorption and storage of most LiPSs in the proposed QCCS; besides, a great many large pores can still be detected, indicating the preservation of abundant ion pathways after long cycle periods. While for the CF interlayer, only quite small amounts of sulfur species can be found on the carbon fibers due to their weak interactions with the LiPSs. Moreover, the insert in Figure 6 also shows the severe anode corrosions with many visible small tubers on lithium foil surface for the CF cell, which can be originated from the diffusion of soluble polysulfides that react with the lithium metal. In vivid contrast, the lithium anode in the V-CF cell exhibited a relatively uniform and smooth surface after cycling. All these investigations indicated the advantages of the architecture-derived enhancement on the electrochemical stability, by introducing near-vertically aligned V_2_O_5_ nanoplates on carbon fiber surfaces to form efficient interlayer for high-performance Li–S batteries. 

## 4. Conclusions

In summary, a novel near-vertically aligned V_2_O_5_ nanoplates anchored carbon nanofiber interlayer was elaborately designed and synthesized, which showed significantly enhanced stability of electrochemical redox for Li–S batteries. Using the special V-CF composite structure, an effective “quasi-confined cushion space” that can flexibly accommodate the utilization of LiPSs and prompt the construction of stable electron/ion transportation pathway was demonstrated. For the Li–S battery assembled with the V-CF interlayer, a high initial capacity of 1140.8 mAh·g^−1^ and a stable reversed capacitance of 1110.2 mAh·g^−1^ after 100 cycles at 0.2 C were obtained. Additionally, a high reversible capacity of 887.2 mAh·g^−1^ and an ultra-low decay rate of 0.0093% per cycle after 500 cycles at 1 C were also kept, showing great potentials on improvements of the high rate and long cycling stability. The investigations may provide a novel structural-related protocol to address the power loss challenge for Li–S batteries.
